# Edge Based Priority-Aware Dynamic Resource Allocation for Internet of Things Networks

**DOI:** 10.3390/e24111607

**Published:** 2022-11-04

**Authors:** Zulfiqar Ali, Kashif Naseer Qureshi, Kainat Mustafa, Rasool Bukhsh, Sheraz Aslam, Hana Mujlid, Kayhan Zrar Ghafoor

**Affiliations:** 1 Department of Software Engineering, Bahria University, Islamabad 46000, Pakistan; 2 Department of Computer Science, Bahria University, Islamabad 46000, Pakistan; 3 Department of Computer Science, Virtual University of Pakistan, Lahore 54000, Pakistan; 4 Department of Computer Science, COMSATS University Islamabad, Islamabad 44000, Pakistan; 5 Department of Electrical Engineering, Computer Engineering, and Informatics, Cyprus University of Technology, 3036 Limassol, Cyprus; 6 Department of Computer Engineering, Taif University, Taif 21944, Saudi Arabia; 7 Department of Computer Science, Knowledge University, University Park, Kirkuk Road, Erbil 446015, Iraq

**Keywords:** LPWAN, LoRaWAN, QoS, network, scalability, resource allocation, congestion, channel, Internet of Things, 5G

## Abstract

The exponential growth of the edge-based Internet-of-Things (IoT) services and its ecosystems has recently led to a new type of communication network, the Low Power Wide Area Network (LPWAN). This standard enables low-power, long-range, and low-data-rate communications. Long Range Wide Area Network (LoRaWAN) is a recent standard of LPWAN that incorporates LoRa wireless into a networked infrastructure. Consequently, the consumption of smart End Devices (EDs) is a major challenge due to the highly dense network environment characterised by limited battery life, spectrum coverage, and data collisions. Intelligent and efficient service provisioning is an urgent need of a network to streamline the networks and solve these problems. This paper proposes a Dynamic Reinforcement Learning Resource Allocation (DRLRA) approach to allocate efficient resources such as channel, Spreading Factor (SF), and Transmit Power (Tp) to EDs that ultimately improve the performance in terms of consumption and reliability. The proposed model is extensively simulated and evaluated with the currently implemented algorithms such as Adaptive Data Rate (ADR) and Adaptive Priority-aware Resource Allocation (APRA) using standard and advanced evaluation metrics. The proposed work is properly cross validated to show completely unbiased results.

## 1. Introduction 

The requirements for next-generation communication systems are high-speed data transmissions using 5G and 6G data communication standards [1]. One of the most important requirements for a 5G network is long battery life of the EDs and seamless integration with Internet-of-Things (IoT) networks. Some other important challenges such as scalability, cost efficiency, battery life, processing power, indoor coverage, throughput, and persistent connectivity need to be considered to improve IoT network services and enhance quality. The term IoT is commonly used to specify various standards and research areas used to access real physical objects. Several characteristics are required for data communications in these networks, such as long or short range, low bandwidth, and the ability to connect a large number of end devices (EDs) [2,3].

Some of the most commonly used IoT technologies are Radio Frequency Identifiers (RFID) [4,5], limited-range technologies (NFC, Bluetooth, and ZigBee), Wireless Sensor Networks (WSN), and cellular technology (2G, 3G, 4G) [6]. Several LPWAN standards such as Sigfox, Weightless, NB-IoT [7], and Low Power Wide Area Network (LoRaWAN) are currently used to meet the requirements of different IoT applications. LoRaWAN is considered as a competitive technology for various IoT networks. Moreover, LoRaWAN relay devices can extract data from thousands of IoT sensor nodes over a considerable range in kilometers. The massive connection of IoT devices with Base Station (BS) can have a negative impact on signal strength and control messages. For these reasons, current cellular network technologies are not suitable to fully support the envisioned IoT networks [8]. Another technology known as LPWAN has been launched as a result of the rapid growth in the number of connected devices; this technology is best suited for networks with high levels of connectivity [9]. In single hop technologies, EDs are directly connected to the gateway, which further sends packets to a network server. Several applications are using LPWAN technologies such as smart city applications, wearable (personal) IoT applications, consumer applications, smart metering, logistics, industrial monitoring, and agriculture monitoring applications.

Energy consumption of LoRa-enabled smart EDs is a major challenge due to the highly dense wireless environment, limited battery life of LoRa EDs, coverage, interference, and number of collisions [10]. All these possible QoS parameters drastically increase delay of LoRa-enabled terminals which contribute towards high consumption. Using the current LoRa framework for IoT applications, a huge amount of data transmitted towards gateway, resulting in real-time Packet Error Rate (PER), low throughput, high number of collisions, and retransmissions. All of these issues contribute significantly to transmission delay and consumption. Intelligent, QoS-aware, and efficient service provisioning is urgently needed to better address this problem, which directly affects QoS of such networks.

In this research, we provide a novel IoT-based approach to determine the best solution for applications requiring smart health monitoring, which addresses the issues of energy consumption and network capacity and improves performance by allocating efficient resources to EDs. The smart sensors used in this paper are smart blood pressure, heart rate, and pulse oximeters. These sensors continuously generate large amount of data to LoRa gateway (GW), which they forward to the network server (NS). Due to its long range, low cost, and efficient obstacle penetration (CSS modulation), LoRa network is one of the best choices for smart health monitoring applications. 

To achieve the best performance from LoRa EDs, it is very important to choose appropriate transmit power, bandwidth, and SF for the terminals. Another factor that plays an important role is the distance between the smart EDs and the forwarding devices, called gateways. By Increasing the distance between EDs and gateways, efficient mechanism of transmit power must be considered. LoRaWAN solves these problems through ADR, but to keep the complexity as low as possible, LoRa ADR allocates resources in a network environment where we have a limited number of smart nodes. The number of received packets is increased by conventional ADR for class A EDs but ultimately this enhances energy consumption as well. To reduce energy consumption, we propose to integrate dynamic reinforcement learning into the LoRa network. As we know, all attributes of EDs sending packets to gateways are received by a central GW. Therefore, in our case GW runs a dynamic reinforcement learning algorithm to update the parameters such as transmit power, SF, BW, and channel for ED. The main contributions of the proposed technique are:

(i) Gaussian Mixture Model (GMM) is used for profiling, and after profiling we not only consider PSR and PER, but also optimize the energy consumption of EDs.
(ii) After assigning EDs to profiles such as HPP or MPP, dynamic reinforcement learning algorithm extract the current state of all configured EDs. Appropriate actions are performed by resource learning agents according to the optimized policy to obtain a refined Reward.
(iii) Finally, resource learning agents assign updated and optimized parameters to EDs accordingly.

The rest of the paper is organized as follows: Section 2 presents the related work. Section 3 illustrates the system model and its formulations. Section 4 presents the results and discussions. The last section concludes the paper with a future direction.

## 2. Related Work

This section highlights the articles that target the enhancement of network performance in terms of energy consumption. The authors in [11] evaluated the performance of LoRaWAN in an indoor environment. The main focus of researchers in this article; is to determine the strength of LoRa network signals in an undesirable environment. Other parameters such as collisions, success rate, delay, and energy were investigated. The authors also analyzed the integration of LoRaWAN with 5G. The network was established using a server, a gateway, and a terminal device. The quality of the received signal was measured from different locations to cover an entire building. It was found that the signal quality was not affected by walls between the rooms and the laboratory. Only the affected area in the basement was impaired enough to contribute to losses. The authors in [12], provide mathematical modeling to evaluate the factor of re-transmissions in LoRa network. Intelligent, QoS-aware, and efficient service provisioning techniques are used by authors specifically to address the issues, which directly affects QoS of LoRa networks. In [13], the authors discussed issues such as collisions, throughput, and consumption to provide sufficient solutions to improve the performance of LoRaWAN. The researchers in [14] presented the comparison of LPWAN standards and focused mainly on the LoRa standard. In [15], authors improved the performance of the LoRa network in terms of throughput by allocating appropriate resources. Further allocation of the same parameters to multiple nodes may increase the loss ratio in the densely populated environment. In [16], the authors described infrastructure-based solutions for smart applications. They also presented the various performance parameters of the LoRa network such as collisions and interference. Several components of LoRaWAN architecture were thoroughly discussed and elaborated. Various tests and simulations were performed to analyze the QoS parameters.

In [17], the authors discussed many factors affecting the number of collisions that cannot be solved by conventional time series analysis algorithms. Therefore, deep learning methods were applied to predict the collisions by analyzing these factors in an LPWAN system. In this paper, a Long Short-Term Memory Extended Kalman Filter (LSTMEKF) model was proposed for collision prediction in LPWAN considering temporal correlation, which can improve LSTM performance. In [18], the authors elaborated different approaches for large-scale smart device connectivity. They also discussed the advantages and disadvantages of smart devices and their design aspects, especially with respect to smart applications in urban areas. Authors in [19], implements Slotted Aloha in LoRaWAN environment to evaluate the effect of number of collisions, packet success rate, throughput, delay and energy consumption. Slotted Aloha in LoRaWAN somehow improve the performance of LoRa network in terms of collision and throughput but delay factor is on a higher side. 

In [20], authors use 3D Scattering model to evaluate propagation path delay in LoRaWAN environment. The article largely contributes by investigating the propagation path delay experienced by LoRaWAN under 3D semi-ellipsoid model. Similar to the previous mechanism in [21], the authors investigated the failure probability of Aloha-based access under the Bianchi model. The authors in [22], analyzed the collision probability of Aloha by using the stochastic geometry approach. Furthermore, they also analyzed the maximum load capacity under various packet loss rates.

In [23], the authors suggested LoRaWAN for smart health monitoring applications. The article mainly focused on monitoring blood pressure, glucose, and temperature in a rural area. The main motivation of the paper is to reduce the burden of long trips for people living in remote areas, to visit hospitals, while minimizing the communication cost. Moreover, the results demonstrated that the power consumption of our monitoring system is at least ten times lower than other long range cellular solutions, such as GPRS/3G/4G. In [24], the authors proposed a system that focuses mainly on two tasks: first, on monitoring the status of miners, and second, on overall monitoring. Semiconductor gas sensors were used to monitor the level of unsafe gasses. The microcontroller sounds an alarm to the person through a buzzer when the level of a smoke sensor exceeds the threshold and sends the information to the monitoring region through the LoRaWAN module. There are a number of reasons why miners can collapse and lose consciousness while working underground. The system employs the LoRaWAN module to send a crisis warning to the supervisor whenever someone falls somewhere for any cause in order to solve this issue. An intelligent alarm system is installed for the miners’ safety to warn them in time to escape the mine in an emergency. Using LoRaWAN technology, this system continuously scans the mine and warns the workers and the appropriate person from the ground station. Thus, the proposed system reduces the mortality rate and disease alerts for the workers in the mining industry. 

Authors in article [25] assessed the functionality of a LoRa network with numerous smart EDs. Through a dependable network, these endpoint devices link patients, nurses, and medical professionals. In this study, we investigate several LoRaWAN protocol elements that significantly affect power usage and transmission latency. Additionally, based on software simulations, our LoRa-based network implementation appears to be a viable choice for enabling strong, dependable, and affordable IoT implementation with modest bandwidth needs. 

Table 1 presents several research papers and highlights the objectives in terms of application requirement, SF, BW, Tp and energy consumption. The researchers in [26] proposed an unsupervised learning approach to prioritize packets at different levels. On average, 1000 smart nodes send data to the gateway. K-means was used as an unsupervised method to extract different clusters based on the measurements received from smart applications such as humidity and weather temperature. Various weights were calculated based on the readings received from the smart nodes at the gateway. These weights help to place the smart nodes in different clusters. Overall, this approach works well to improve performance in terms of delay and energy. The priority scheduling algorithm PST was used and the result shows that it significantly reduces delay and consumption. In this paper [27], author’s present mathematical models that performs characterization of LoRa enabled smart nodes current consumption and energy cost of transmitted packets. The models, which have been derived based on measurements on a currently prevalent LoRaWAN hardware platform, allow us to compute the impact of relevant physical and Medium Access Control (MAC) layer LoRaWAN parameters, as well as error rate and collisions, on the basis of energy performance.

In [28], the authors used resource scheduling algorithms to mitigate the delay in wireless communication. The authors used banker’s algorithms to manage resources efficiently in this study. The execution time of this algorithm was also taken into account for fair allocation of resources. The authors in [29] discussed that wireless communication networks reduce the overall cost of deployment and increase flexibility. However, all these benefits come at a cost of high delay probability and loss of packets. This study mainly focused on modeling LoRaWAN as an event-triggered modeling scheme in Matlab.

The authors in [30] proposed a routing-based profiling algorithm in which end devices are distributed non-uniformly. They further assumed that there are different number of end devices in each profile (cluster). A stochastic model was implemented in this research to know about the consumption of nodes in a multi-hop environment. A cross-layer optimization protocol called Adaptive Transmission Power Control-Based Reliable Data Forwarding (APRF) scheme was put forth by the authors in [31] and uses broadcast technology to increase network dependability, decrease communication delay, and ultimately improve performance in terms of energy consumption. 

Furthermore, the authors in [32] proposed two different spreading factor (SF) allocation techniques, EXPLoRa-SF and EXPLoRa-TA. These schemes provide low interference in a cluster-based environment with enhanced time-on-air (ToA). Additionally, EXPLoRa-SF algorithm assigns the same SF and performs successful transmission without any collision. The simulation suggests that the high value of SF provides long coverage but sometimes they contribute to a high number of collisions. To increase LoRaWAN scalability and energy consumption in a dense IoT environment, the authors of [33] presented an adaptive priority-aware resource allocation approach. In [34], authors evaluate the coverage of LPWAN technology via real-life measurements. The experiments were conducted in the city of Oulu, Finland, using the commercially available hardware devices. Authors observed the maximum communication range of over 15 km on ground and close to 30 km on water. 

In [35], the authors performed network slicing in LoRa networks using different slicing strategies as well as different distributions of the spreading factor. They used an adaptive dynamic inter-slice resource reservation algorithm based on maximum likelihood estimation that avoids resource starvation and prioritizes one slice over another depending on urgency and reliability. In addition, a novel intra-slicing strategy was evaluated that maximizes the efficiency of resource allocation in each slice with respect to its QoS requirements. Simulation results conducted in realistic IoT scenarios highlight the utility of our proposal in improving the consumption and delay of IoT devices.

The main goal of dynamic resource allocation is to improve QoS in terms of energy consumption. First, a machine learning approach, Gaussian mixture model (GMM), is applied to GW to create an optimal number of profiles. Then, the optimized resources (channel, spreading factor, and transmit power) are allocated to the terminals to improve the performance in terms of reliability and consumption. After thorough investigation, it is concluded that few studies provide energy efficiency by fine-tuning the transmit power (T_P_) or allocating the Spreading Factor (SF). To the best of our knowledge, only priority-aware dynamic resource allocation with adaptive congestion control at the profile level provides optimal results in terms of network capacity and reliability.

## 3. System Model and Formulations

The system model depicts a scenario for smart health monitoring in residential areas, e.g., smart pulse oximeter, blood pressure, and heart rate. The said scenario is implemented using two gateways *(GW’s)* and all nodes are randomly distributed over an area of 5 km^2^. All the end devices *(EDj)* are initially configured using LoRa model SX1272, where *j* is 1 ≤ *j* ≤ 3000. All *ED_j_* are static and use the class A specification. Class A *EDs* use two receive windows (Rx1 and Rx2) for responses from the gateway in case of an acknowledged communication [18].

### 3.1. Profiling with Gaussian Mixture Model 

**Figure 1:**
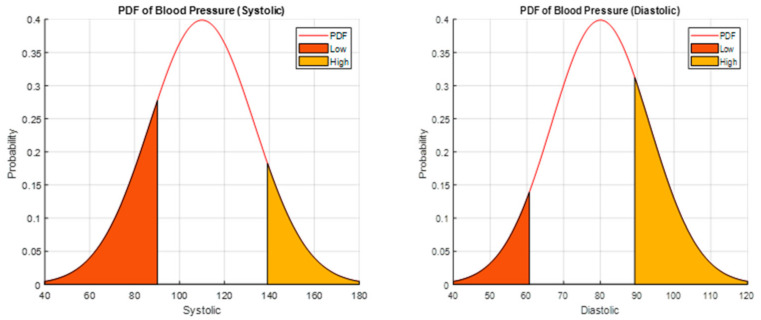
Gaussian distribution of Smart Blood Pressure Wearable in Terms of Systolic and Diastolic.

The GMM with K-means algorithm is implemented on the gateway in the LoRaWAN environment. After the optimal profiles are defined by the GMM algorithm, the intelligent *EDs* are assigned to each profile according to the probability density function. Based on the Adaptive Scheduling Algorithm (ASA), the traffic from different profiles is prioritized according to Low Priority Profile (LPP), Middle Priority Profile (MPP), and High Priority Profile (HPP). An example of smart blood pressure readings in form of systolic and diastolic are presented through Gaussian distribution in Figure 1.

$P_{x}\left(x\right)$ is the probability density function representing patients lying in different regions depends on readings. Moreover, μ, σ, and $\sigma^{2}$ are terminologies depicting the average (mean), standard deviation, and variance of the bell shape curve. Equation (1) shows the probability distribution function for one-dimensional data readings of patients.

(1) $$P_{x}\left(x\right)=\frac{1}{\sqrt{2\pi \sigma^{2}}}\;e^{-\frac{{\left(x-\mu \right)}^{2}}{2\sigma^{2}}}$$

Mathematically, Gaussian distribution of *EDs* or its vector representation becomes *X =* {*x*_1_, *x*_2_, *x*_3_, ... *x_n_*} ∈ *R*^3^. 

To know about profile *k* of *x_i_*, *z_i_*_│*W*_ is nearly equal to categorical (*w*), which means:

(2) $$P(Z_{i}=k|w)=W_{k}$$

$P\left(Z_{i}=k|w\right)$ means that *ED_i_* belongs to profile *k*. *W_k_* is a mixture weight of *k* which is equal to 1 if the value of mixture is between 0 and 1.

(3) $$\sum_{k}W_{k}=1,\;\mathrm{if}\;0\leq W_{k}\leq 1$$

As each profile has its own center and co-variance, where *X_i_* is generated from probability distribution as shown below:

(4) $$X_{i}\;|Z_{i}=k\;\sim \;N\left({µ}_{k},\sum_{k}\right)$$

${µ}_{k}$ is considered as profile center and $\sum_{k}$ is the co-variance of profile.

Given the profile center and its co-variance, we can compute probability *P* for specific value of *X_i_*:

(5) $$P(X_{i}=x_{i}\;\;|\;\;{µ}_{k},\sum_{k})$$

**Figure 2:**
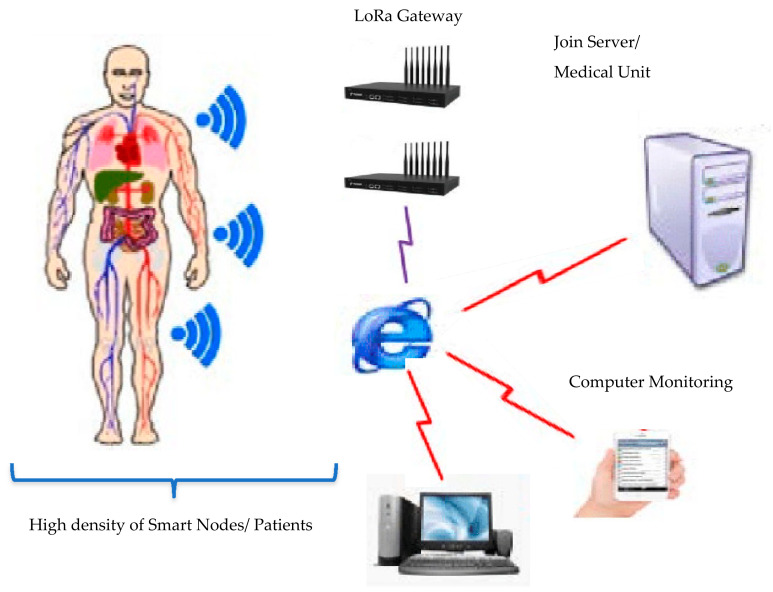
Smart Health monitoring LoRa network.

Figure 2 shows the system model for smart health monitoring scenario covering residential area of 5 to 6 Km^2^.

(6) $$P\left(x/c\right)=\frac{1}{\sqrt{2\pi \;\left|\sum c\right|}}.\;e^{-\frac{1}{2}\;{\left(x_{i}-{µ}_{c}\right)}^{T}\sum_{c}^{-1}\left(x_{i}-{µ}_{c}\right)\;}$$

$P\left(x/c\right)$ is probability density function of *i*th node with respect to center point of profiles *c*. After this, in exponential component, we are subtracting mean component from the *i*th instance of end devices ${\left(x_{i}-{µ}_{c}\right)}^{T}$ and in the middle we are multiplying it by inverse of co-variance $\sum_{c}^{-1}\left(x_{i}-{µ}_{c}\right)$. The co-variance component describes the shape of Gaussian distribution.

To handle such sensitive patient data, we need to deploy two gateways in our LoRa network. Sometimes multiple *GWs* increase the interference [38], but we need to deploy these *GWs* at points where the interference between *EDs* will be as negligible as possible. With this large number of *EDs* sending to the *GW*, the biggest challenge is keeping the packet acceptance rate high. Achieving high throughput means a low number of retransmissions, which drastically reduce the consumption of the sending *EDs*.

### 3.2. Reinforcement Learning through RLA

Reinforcement learning (RL) algorithms are based on reward *R* and policy *π*. In RL, we have certain agents. These agents perform actions *A* based on the current state *S*. After they perform an action *A*, they receive a reward *R*. Policy is a kind of function that determines what actions should be taken in different states *S*. We will figure out and optimize this function so that the agent gets maximum *R*. Reward is a function *F* depends on *S* and action *A* it takes: Reward: *F: S* ∗ *A → R* [41].

The goal of the policy derivation algorithm is to allocate the most appropriate resources to *EDs* that will ultimately help them consume less energy. This can be translated in RL jargon as determining the optimal action *a* (from a set of *A* admissible actions, i.e., *a* ∈ *A*) for a state *S* (with *s* ∈ *S*). When an action is performed in *S*, a reward is obtained. The reward consists essentially of updated configuration parameters such as *(DR*, *SF*, *Tp*, and *BW)*. The function ℛ defines such a reward mathematically as a mapping between state-action pairs and real numbers, i.e., ℛ: *S × A →* ℝ.

The RLA is in charge of distributing these updated resources to EDs after obtaining the incentive. To enable RL entities to likely shift from one state to another, these transitions can be stochastic. That is, *P(S′|s*, *a)* is the probability of the next state *S′* after action *a* is executed according to the strategy.

To find an optimal action policy, i.e., *Policy: π: (S → A)* that maximizes the expected total reward over a finite time horizon:

*V_π_(S_t_)* = *E* [*R_t+*1*_* + ∂ *R_t+*2*_* + ∂^2^*R_t+*3*_* + …….], 0 ≤ ∂ < 1 (7)

where *∂* is the weighted value or discount rate. In practice, we are more interested in the immediate reward, so we need *∂* = 0 to reduce the impact of other rewards.

(8) $$V_{\pi }(St)\;=\;E\left(\sum_{i=1}^{\infty }\partial^{i-1}R_{t+i}\right)$$

The main focus of this manuscript is to dynamically allocate resources through reinforcement learning [41] based on DRLRA. The major purpose of this method is to assign the EDs with effective resources, which ultimately improves performance in terms of energy usage. A total of 3000 intelligent *EDs* are deployed and considered. After profiling through GMM (described earlier), *EDs* are assigned to HPP or MPP or LPP through resource learning agents (RLAs). Now, we need to implement RLAs on *GW* for the number of *EDs* in the LoRa network. The reason for using different RLAs for a set of *EDs* in the selected profile is that GMM basically performs soft profiling, which means that sometimes we have *EDs* in the same profile that are far apart due to their elliptical shape. Therefore, *EDs* that are close to each other in the same profile are assigned to a single RLA. The RLA gathers data on the EDs’ present state S and takes specific actions A based on an ideal strategy. After the action *A*, the reward *R* is calculated and the updated parameters (*DR*, *SF*, *Tp*, *BW*, *Channel freq,* and *CR*) are assigned to a group of smart *Eds*. *Eds* that are not far from each other may need the same parameters as *DR*, *SF*, *Tp*, *BW*, and *Channel freq*. To avoid collisions, we assign different coding rates (*CR*) to *EDs* managed by a single RLA.

The smart health monitoring network is based on 3000 end devices *(ED_i_)* where *i* ∈ 1, 2, 3, …. 3000), deployed in a densely populated area. All the *ED_i_* and *GW* are randomly deployed on a certain location, and we can identify these *Ed_i_* based on their geographical coordinates. Moreover, the location of *ED_i_* is represented as *(x_i_*, *y_i_*, *z_i_)* in the geographical area. LoRaWAN is a single-hop network between *ED_i_* and *GW*. The communication between *EDi* and *GW* is accomplished with the help of several channels and these channels are dynamically assigned to *EDi* based on traffic. First of all, *GW* is responsible for making profiles by using a probabilistic approach, known as GMM. It is used to design optimum number of profiles. After the number of profiles are decided the *GW* assignes different priorities *P_r_* to profiles. Priorities are set for each profile so that we transmit only critical readings from *EDi*, to intelligently manage traffic. In this way, network capacity is intelligently managed and performance in terms of energy consumption is also improved. After the first reading transmitted to *GW* by an *EDi*, the *EDi* now follow the extended Aloha, which states that the next reading is transmitted only if it differs by +/−5% from the previous reading. The state of *EDi* that need to be maintained by RLA contains information of distance *(d)*, ToA (extracted from current *SF*), current *SF*, *T_p_* associated with *EDi*, received signal strength *(RSS)* at *GW*, and current channel usage by *ED_j_* in percentage (%). Now, the RLA calculates reward, and assigns updated parameters to *EDi*. 

The resources such as transmit power and *SF* are allocated by Adaptive Data Rate (ADR) in a conventional LoRa network [14]. In smart health monitoring scenario, *EDs* from HPP transmit packets towards *GW* for 15 min or 900,000 milliseconds at maximum. After 15 min, *Eds* from MPP are allowed to transmit readings towards *GW* for 5 min or 300,000 milliseconds. The RLA is designed for set of *Eds* on *GW*, which automatically updates the allocated parameters for *Eds* according to requirement. The major goal of this study is to evaluate how the DRLRAP affects the energy usage of the LoRa network.

**Figure 3:**
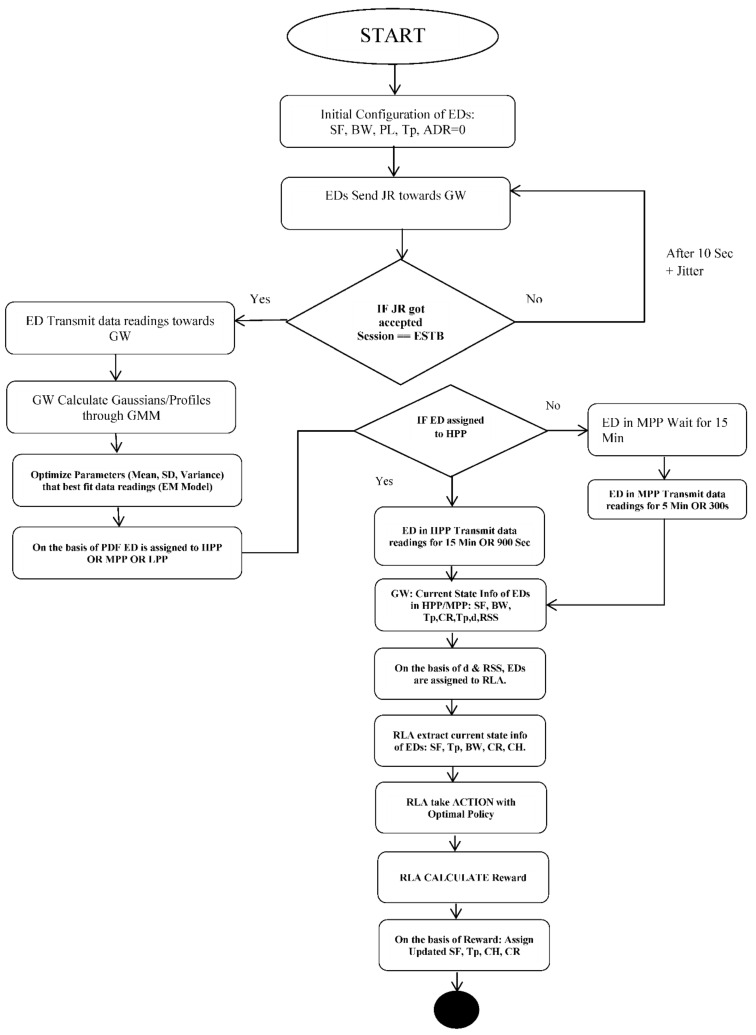

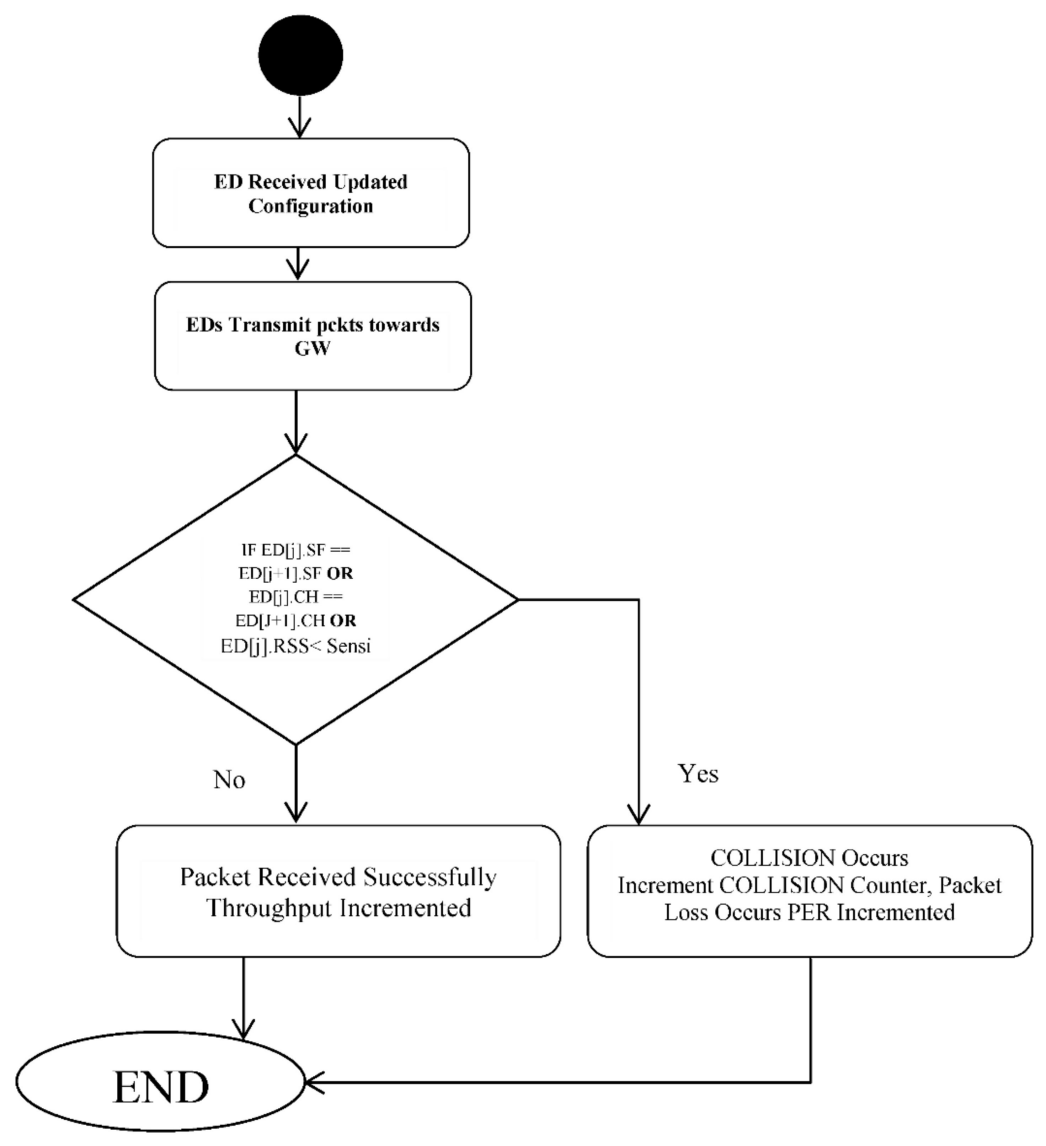
RLA Procedure in DRLRA.

Figure 3 shows the flow of Dynamic Reinforcement Learning Resource Allocation Profiling with Resource Learning Agents.

The main parameters affecting the performance of the LoRaWAN network are *SF*, *TP*, *BW*, and channel attributes. Therefore, the dynamic RLA must be well equipped in terms of learning before allocating resources to *EDs*. The data channels used by the LoRa network are 868 MHz and 6 *SF* from 7 to 12 are used. *T_P_* used in our model is from 2 dbm to 14 dB with a spacing of 2 dbm. 

Based on collected parameters, dynamic RLA responded with a reward in terms of updated configuration. The reward according to corresponding actions is calculated as in Equation (9):

(9) $$EDr=c\;\frac{\sum_{i=0}^{N}F_{i}}{\sum_{i=0}^{N}E_{i}}$$

where, *N* is the number of *EDs*, *F* is the total number of frames received at the *GW* for a specific duration, and *E* is the total energy consumed during the active duration of *ED*. According to the Equation (9), the reward for concerned *ED_r_* increases with the increase in number of the total number of frames. With the increase in consumption of energy, the reward for *ED_r_* decreases. The reward *r* is automatically varying with the change that occurs in the state of *ED*. To optimize the reward, we have to give priority to the success rate of frames by multiplying it with term *c*. Algorithm 1 presents the overall flow of DRLRAP.


**Algorithm 1:** DRLRAP based on GMM Profiling.Declare variables: Ed_i_, distance (d), Initial SF, Initial DR, Initial T_p,_ BW, ToA, Channel Usage (CH_US), (ED_j_)_Pr,_ P_r_
To mitigate Energy Consumption, Delay: **START LOOP for** ED_i_ **do** 1.     ***if*   ** ED_i_ BELONGS TO **HPP** OR ED_i_ with Maximum value (Pr) in **HPP**
2.              Initially, **ED_i_** transmits packets at a Maximum value of **SF** i.e.,               **SF12 & TP = 14 dBm.** 3.              Dynamic RL defines Groups inside HPP based on d and RSS. 4.              Design RLA for each Group inside HPP. 5.              RLA checks EDi State takes Action and calculates Reward. 6.              RLA use Q Function to calculate future Reward. 7.     At GATEWAY 8.     ***if*  RSS** of **ED_i_** < **SENSI_EDi, SFi_  AND CH_US _EDi_** > 70%     (**CH_US _EDi_** by ALCAA Algorithm) 9.              Then Perform 10.              ***if* SF_EDi_** is 12, Keep it the same, 11.               ***else* DECREASE SF_EDi_** by 1. 12.               **UPDATE ED_i_** with new **SF_i_, BW**, and **DR_i_.** 13.         At **GW_j_**, **REPEAT** 14.     ***if*  RSS** of **ED_i_** >= **SENSI_EDi, SFi_  AND CH_US _EDi_** > 50% 15.              Then Perform 16.              ***if* SF_EDi_** is 12, **DECREASE SF_EDi_** by 1. (**INCREASE DR**) 17.              **UPDATE ED_i_** with new **SF_i_, BW**, **DR_i,_** and Adjust **TP_EDi_** 18.              **Set RSS_Thresh_** and **TP_V_** (TP Inc/Dec value) 19.      **REPEAT** 20.     ***if*  SENSI_SF EDi_ > RSS_Thresh_** 21.              **TP= TP − TP_V_** 22.              **UPDATE ED_i_** with new **SF_i_, BW**, **DR_i,_** and Adjust **TP_Edi_**


The SX1272/73 works at 3.3 V per the LoRaWAN specification document [5], and the current consumptions in idle, transmit (at 20 dBm), and receive states are, respectively, *I_idle_* = 1.5 A, *IT_x_* = 125 mA, and *IR_x_* = 10.5 mA [10]. The voltage *V_p_*, the current, and the length of time that the corresponding state lasts are multiplied to determine the energy consumption. For instance, the energy consumption E cons for a transmission when an *ED* is in the transmitting state is: *V_p_*⋆*IT_x_*⋆*ToA.* If no data is being transmitted or the receiving windows are not open, an *ED* will be in an idle state.

(10) $$\;\;\;\;\;\;\;\;\;\;\;\;\;E_{cons}=\sum_{i}\sum_{packets}\left(V\right)⋆\left(I\right)⋆(ToA)$$

The current consumption will be *IR_x_* after an *ED* opens the receiving windows and enters the idle listening mode. The *ED* enters receiving mode and the current consumption stays *IR_x_* if the Ack preamble is detected.

Table 2 shows the sensitivity according to DR, SF, and BW for the SX1272 LoRa module.

## 4. Results and Discussions

This section is dedicated to the simulation results targeting *PSR*, *PER*, and *EDs* consumption. *GW_j_* assign three different priorities *P_r_* (HPP, MPP, and LPP) to the *Profs* so that different simulations are performed to determine the behavior of *ED_i_* in terms of Packet Success Ratio (PSR), Packet Error Rate (PER), and collisions. The normalized values of PSR, PER, and collisions are calculated for all profs. The simulation performed two GWj in the scenario we created, so we have two HPP at the same time. The reason for selecting HPP individually for each *GW_j_* is that we can have critical values from other *ED_i_*. To account for the severity of patients in the smart health monitoring scenario, we want as many *ED_i_* as possible to be able to send data simultaneously, while also keeping QoS in mind.

The results of DRLRA are extensively compared with ADR and APRA schemes for resource allocation. We used Python for the implementation of LoRaWAN in the smart health monitoring scenario. The simulation is performed using well-known libraries of python used to create an *EDs*, agents, gateways, etc. The idea behind using Python libraries is to create an environment that is flexible enough to control and manage all network-related functions. Different objects are assembled and configured as well as scheduled for certain discrete events. Table 3 provides all the details about parameters used during the simulation.

**Figure 4:**
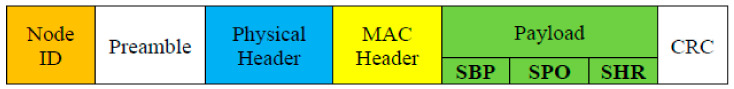
20 Bytes Packet Structure.

One major assumption is the static nature of LoRa *EDs*. Due to its static nature, *EDs* that are far from the *GW* need more transmit power to perform successful transmission. This leads to the high energy consumption of *EDs*. Another limitation is the absence of a dedicated LoRa simulator available in the market. Further, multiple *GWs* increase throughput but it also contributes towards interference [38] and cost factors. In our case, we are using GMM with K-means probabilistic approach to create profiles that ultimately mitigate number of *EDs* per profile. This increases throughput as the number of collisions automatically decreased with the small number of *EDs* transmitting data at one time. The structure of the complete packet used in the simulation is shown in Figure 4.

*GW_j_* assigns three different priorities *P_r_* (HPP, MPP, and LPP) to *prof*. Different simulations are performed to know the behavior of *ED_i_* in terms of *PSR*, *PER*, and collisions. The normalized values of *PSR*, *PER*, and collisions are calculated for all *prof*. The simulation carried out two *GW_j_* means at one time we have two HPP. The reason to choose HPP for each *GW_j_* individually is that we may have critical readings from other *ED_i_* as well. After both the *GW_j_* select their HPP and MPP profiles, now the *ED_i_* in HPP will be allowed to transmit packets. The behavior of PSR with a varying number of nodes will be rigorously analyzed. The packet size for our simulation environment is 20 bytes.

**Figure 5:**
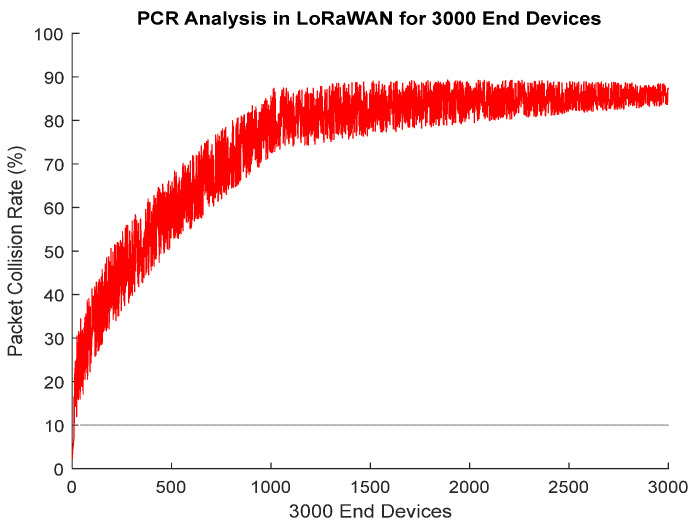
PCR Analysis in LoRaWAN for 3000 End Devices.

In the simulation environment, there are 1000 patients in total, but each of these patients is equipped with three different smart LoRa-enabled wearables (smart blood pressure monitoring, smart pulse oximeter, and smart heart rate monitoring). Figure 5 shows the behavior of Packet Collision Rate *(PCR)* in percentage (%) having *EDs* on x-axis. In this simulation, a total number of 1000 patients with three smart wearables each transmit data towards *GW_j_*. Initially, *ED_i_* transmits data with SF 12, BW 125 KHz, and *T_p_* 14 dBm. ADR is enabled after the first uplink for all the *ED_i_* in a said geographical area. As with smart health monitoring systems, the *ED_i_* generates a small amount of data, so the payload size is limited to 20 bytes. The behavior for conventional LoRaWAN is presented in Figure 5, which shows a severe increase in *PCR* with the increase of *ED_i_*. So, in the health monitoring scenario, where we have some critical patients, conventional LoRaWAN strongly failed. Figure 5 shows the *PCR* analysis in LoRaWAN for 3000 *EDs*.

**Figure 6:**
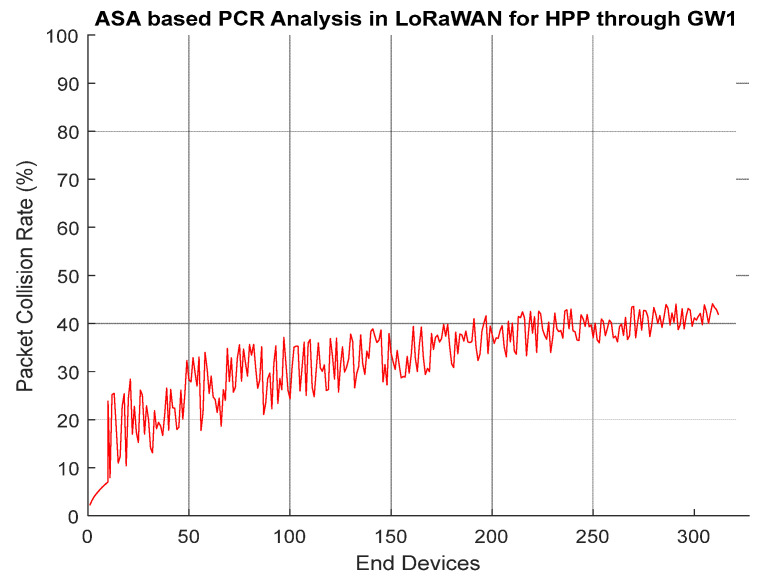
ASA Based PCR Analysis In LoRaWAN For HPP Through GW1.

*ED_i_* in Figure 5 follows Pure Aloha to transmit data towards *GW_j_*. A total of 85% losses are observed in the case of LoRa conventional MAC scheme Pure Aloha. In the case of extended Aloha scheme used in this paper, after sending first uplink packet by an *ED_i_* through Pure Aloha, second packet is forwarded towards *GW_j_* if and only if there is a significant difference between previous and current readings. In this way, unnecessary traffic is blocked, and network capacity is managed efficiently. Figure 6 shows the behavior of *PCR* for HPP. GMM is used to perform profiling based on probabilities assigned to *ED_i_*. After running the simulation, the GMM distributes *ED_i_* into three profiles (HPP, MPP, and LPP). In the first attempt, the 300 *ED_i_* are included in HPP by GMM algorithm. From 3000 *ED_i_*, approximately 300 *ED_i_* are of those with critical readings. After assigning priorities, now all *ED_i_* in HPP is allowed to transmit data towards the designated *GW_j_*.

**Figure 7:**
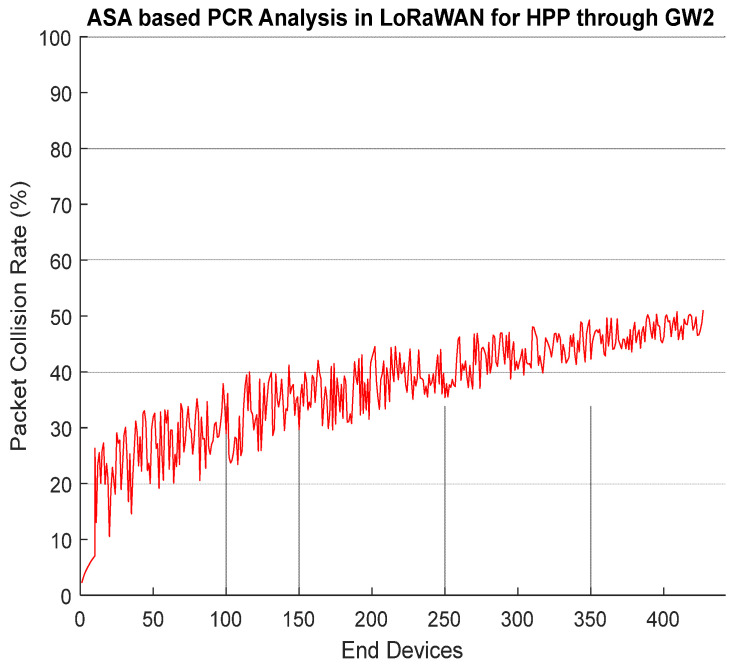
ASA Based PCR Analysis in LoRaWAN for HPP Through GW2.

Figure 7 presents the results of *PCR* through *GW2* having 425 *EDs* (GMM approach running on *GW2* assign 425 *EDs* in HPP based on readings). Now, these 425 *EDs* are on a priority to transmit their frames towards *GW2*. With the increase in the number of *EDs*, the *PCR* ratio is a little bit on a higher side as compared to HPP served by *GW1*.

**Figure 8:**
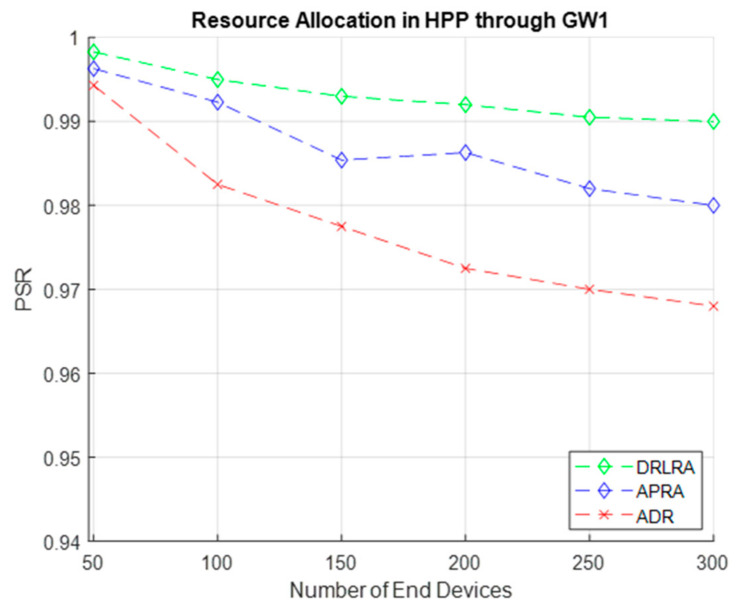
PSR W.R.T DRLRA for HPP and Comparison with ADR and APRA.

With the help of thorough modelling and dynamic resource distribution, the Packet Success Ratio (PSR) for HPP is demonstrated. DRLRA allocates resources such as *SF*, *BW*, channel, and *Tp* on the basis data (readings) received from network environment. Figure 8 demonstrates DRLRA algorithm in terms of *PSR* for HPP. Its comparison with APRA and ADR is also shown in the above simulation. DRLRA outperforms ADR and APRA by 2.2% and 0.975%. *PSR* of about 97% is achieved with the help of profiling and DRLRA algorithm.

**Figure 9:**
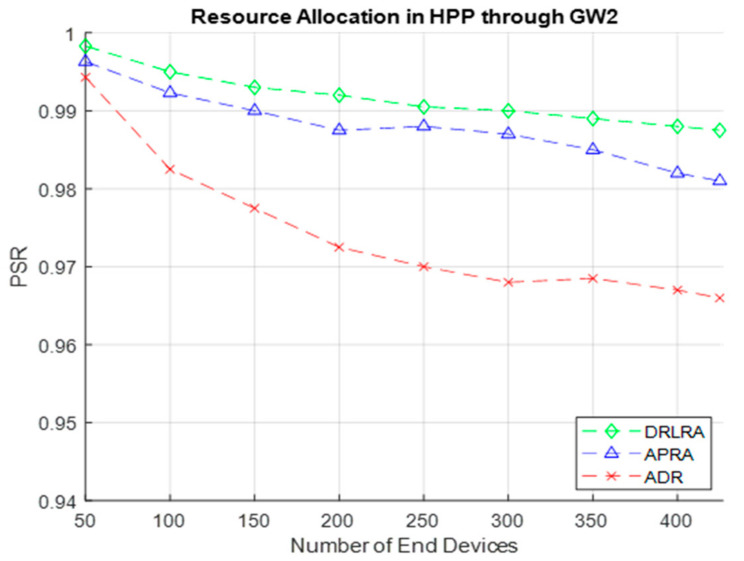
PSR W.R.T DRLRA for HPP and Comparison with ADR and APRA.

Results in Figure 9 show performance of DRLRA algorithm by dynamically allocating resources in HPP through *GW2*. In this simulation, GMM select 425 *EDs* in HPP on the basis of critical readings received. Inside HPP we have several groups, decided on the basis of distance *d* and *RSS*. *RLA* is responsible to assign resources to *EDs* inside the group on the basis of reward. Overall, the performance in terms of *PSR* is enhanced as compared to conventional ADR and APRA by 2.1% and 0.5%. 

**Figure 10:**
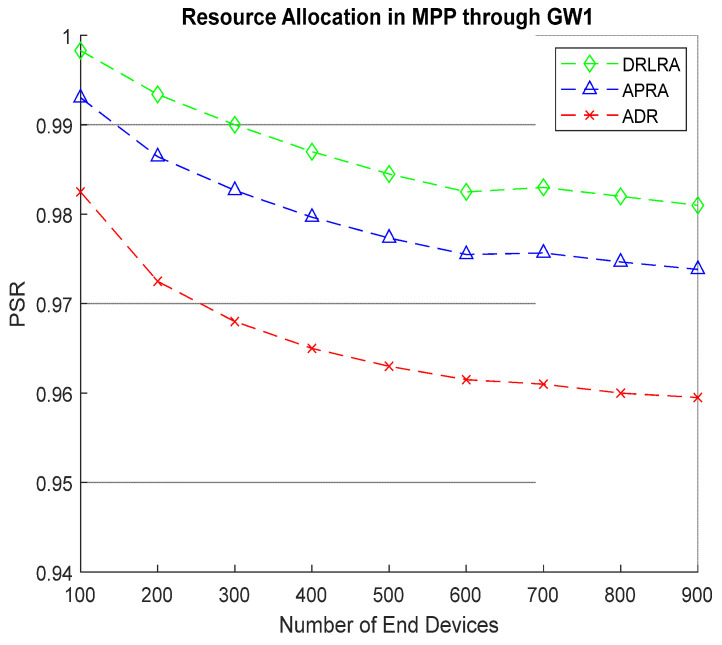
PSR W.R.T DRLRA for MPP and Comparison with ADR and APRA.

Figure 10 shows the behavior of *PSR* for MPP. Almost 900 *EDs* are assigned to MPP depending on the values generated by these smart nodes. The results of *PSR* after allocating resources by DRLRA outperform ADR and APRA by 1.6% and 0.5%.

**Figure 11:**
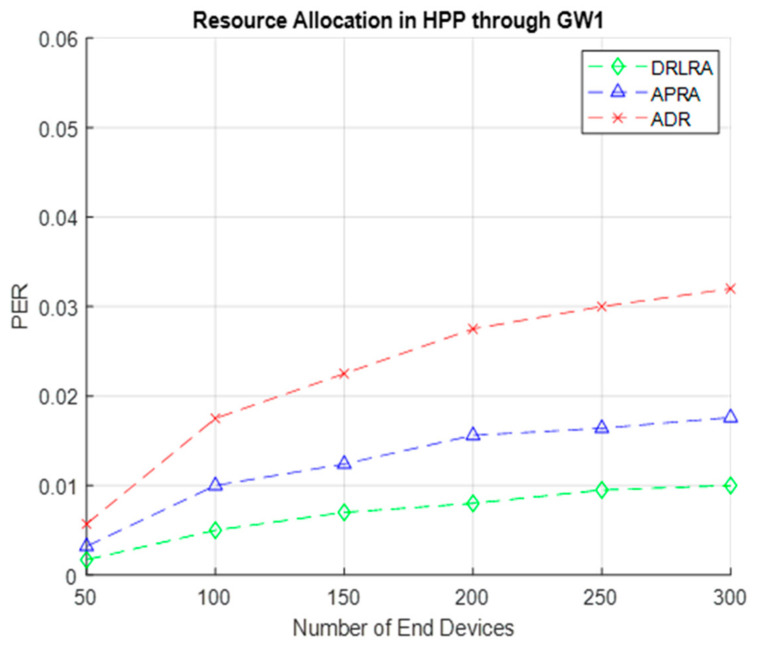
PER W.R.T DRLRA for HPP and Comparison with ADR and APRA.

Figure 11 depicts the performance of LoRa network in terms of *PER* w.r.t number of *EDs*. As in our HPP, we have total of 300 nodes that are transmitting critical readings, so we allocate maximum bandwidth and other parameters by DRLRA. DRLRA profiling algorithm outperforms ADR by increasing *PSR* and mitigating the effect of *PER*. *PER* drastically decreased with the increase in data throughput.

**Figure 12:**
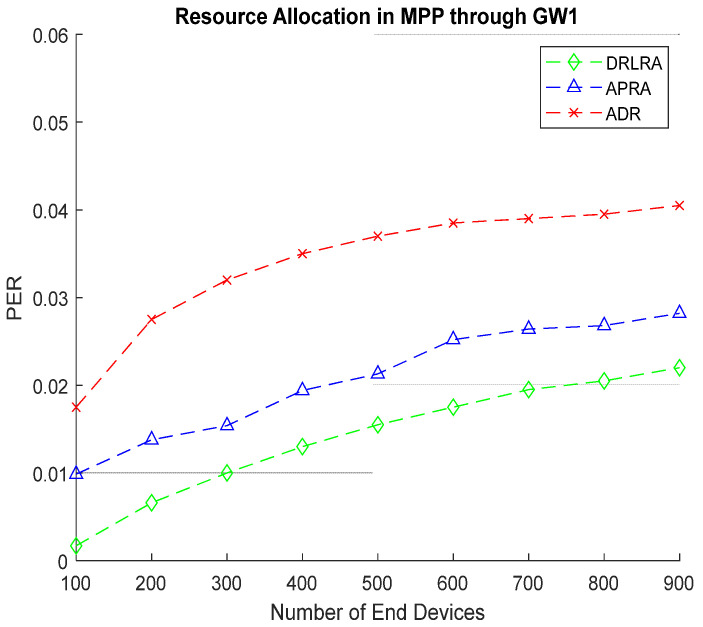
PER W.R.T DRLRA for MPP and Comparison with ADR and APRA.

Figure 12 shows results of *PER* for *EDs* in MPP. Due to increase in the number of *EDs* in MPP, *PER* is little bit on a higher side but still DRLRA profiling outperforms both ADR and APRA by achieving mitigated *PER*.

**Figure 13:**
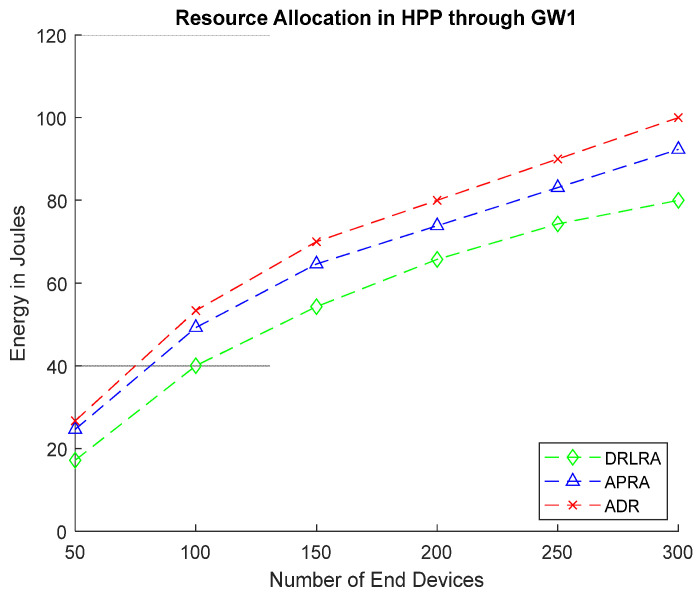
Energy Consumption after Allocating Resources in HPP Through GW1.

Figure 13 depicts the simulation of energy consumption for DRLRA, ADR, and APRA in HPP. To compute energy consumption, it considers several parameters such as current drainage, voltage, processing of packets, and *ToA* of packets transmitted according to *SF*.

**Figure 14:**
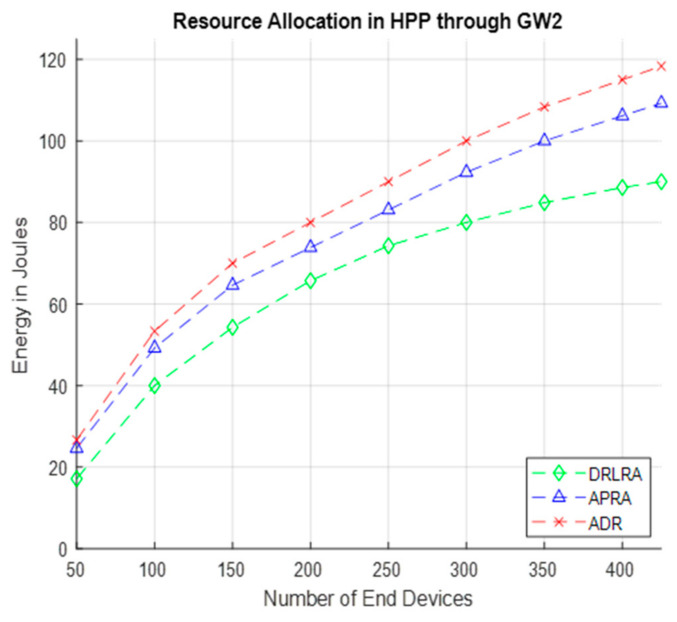
Energy Consumption after Allocating Resources in HPP through GW2.

Figure 14 presents the results of energy consumption for DRLRA, ADR, and APRA in HPP through *GW2*. DRLRA profiling algorithm enhance performance in terms of energy consumption and network capacity.

## 5. Conclusions

LPWAN is the most overwhelming choice for many IoT applications, including to monitor smart homes, smart agriculture, and smart meters. The literature focuses on using Pure Aloha with LoRaWAN to further increase delay with an increase in packet loss. Due to inter-packet arrival and re-transmissions, there has been an increase in delay. However, to achieve optimum performance in LoRaWAN, the delay must be mitigated. To achieve optimum performance in terms of delay, un-supervised probabilistic approach called GMM with K-means is introduced which designs the profiles. Furthermore, ASA is used to prioritize traffic from profiles. The results show that in an environment where thousands of smart *EDs* are transmitting frames, ASA with an un-supervised probabilistic approach drastically mitigates the factor of delay. Another objective regarding the energy consumption of EDs is rigorously analyzed and addressed in the LoRa network. Dynamic reinforcement learning resource allocation is used to allocate resources to *EDs* in different profiles. Inside the profiles, we define different groups based on distance and RSS. This helps RLA to allocate resources inside the group to *EDs* that are far from each other. Furthermore, a comparison with other benchmark resource allocation techniques is also provided. Results of an algorithm for dynamic allocation of resources outperform conventional ADR. The energy consumption is further reduced when we allocate resources by using DRLRA. The out-performance in terms of PER, throughput, collision, and reduced energy consumption can substantially lead towards Green IoT. The future direction of this research is to analyze PPD under a 3D scattering model in LoRaWAN. This analysis may help to decide an optimal placement for gateways for the LoRaWAN scenarios, which may improve the delay experienced by end devices and their energy efficiency.

## Figures and Tables

**Table 1:** Resource Allocation Schemes for a Heterogeneous Scenario.

Research Papers	Publication Year	Objective	Energy	Application Requirements	Spreading Factor (SF)	Bandwidth (BW)	Transmit Power (TP)
[32]	2017	Mitigate the number of collisions and delays	Yes	No	Yes	No	No
[35]	2019	Enhance QoS (reliability, consumption, and delay)	Yes	Yes	Yes	Yes	No
[36]	2018	Analyze unfairness of LoRaWAN in terms of allocation	Yes	No	Yes	Yes	No
[37]	2019	Mitigate the number of collisions	No	Yes	No	No	No
[38]	2016	Analyze the effect of interference.	No	No	Yes	No	No
[39]	2020	Increase utilization of channel and mitigate a collision	Yes	No	Yes	No	No
[40]	2020	Optimize delay and consumption by allocating resources dynamically	Yes	No	Yes	Yes	No

**Table 2:** Sensitivity According to DR, SF, and BW for SX1272 LoRa Module.

Data Rate (DR)	SF with Bandwidth (BW)	Sensitivity of ED w.r.t SF	Bit Rate of Concerned ED
DR5	SF = 7 & BW = 125 Khz	−123 dBm	5470
DR4	SF = 8 & BW = 125 Khz	−126 dBm	3125
DR3	SF = 9 & BW = 125 Khz	−129 dBm	1760
DR2	SF = 10 & BW = 125 Khz	−132 dBm	980
DR1	SF = 11 & BW = 125 Khz	−134 dBm	440
DR0	SF = 12 & BW = 125 Khz	−137 dBm	250

**Table 3:** Parameters Used In Simulation.

Parameters	Values
Application Scenario	Smart Health Monitoring Scenario (SBP, SPO, SHR)
Area	5–10 km^2^
SF	7, 8, 9, 10, 11, 12
BW	125 Khz
Channels	868 Mhz EU Standard
End Devices	1000
Tx Power	2 dBm–17 dBm
ADR	Enabled
No. of Gateways	2
CR	4/5
Packet Size	20 bytes
Optimal Profiles	Prof_k_ = 3
Simulation Times	1 Hour

## Data Availability

Not applicable.
